# P-2180. Linkage to Care, Treatment Initiation, and Outcomes in Individuals with *Hepatitis B Virus* Infection With and Without Cirrhosis

**DOI:** 10.1093/ofid/ofae631.2334

**Published:** 2025-01-29

**Authors:** Adeel A Butt, Peng Yan, Obaid Shaikh, Roger Bedimo

**Affiliations:** Weill Cornell Medicine, Doha, Ad Dawhah, Qatar; VA Pittsburgh Healthcare System, Pittsburgh, Pennsylvania; VA Pittsburgh Healthcare System, Pittsburgh, Pennsylvania; VA North Texas Health Care System, Dallas, TX

## Abstract

**Background:**

Screening for hepatitis B virus (HBV) is far from ideal. Linkage to care is also poor. While traditional models suggest a progression of liver disease from fibrosis to cirrhosis to hepatic decompensation and hepatocellular carcinoma (HCC), emerging evidence suggest that terminal outcomes can occur without preceding cirrhosis. Our aim was to define the cascade of care for HBV and clinical outcomes in HBV infected individuals with and without cirrhosis in the US VA healthcare system.

Annual numbers of individuals with newly diagnosed hepatitis B virus infection and numbers initiated on treatment.

**Figure:**
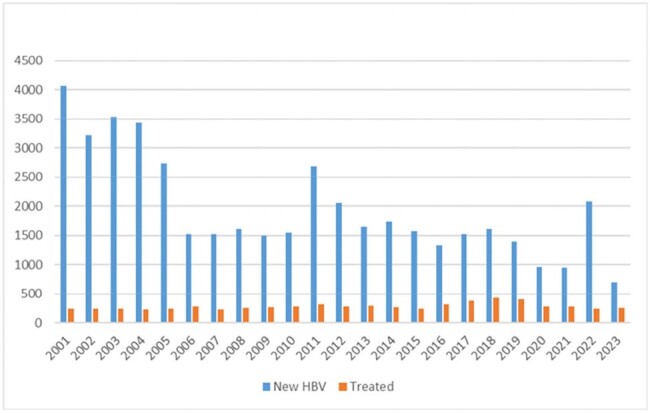

**Methods:**

Within the Veterans Health Administration (VA) national databases, we identified all individuals with a positive HBsAg, HBeAg, or HBV DNA between 2002-2023. We tabulated the number of new infections per year and numbers initiated on treatment. We also calculated the incidence rates for development of cirrhosis, hepatic decompensation (HD), hepatocellular carcinoma (HCC), and death among HBV+ individuals.

Outcomes in individuals with hepatitis B virus infection who were free of cirrhosis at baseline.

**Figure:**
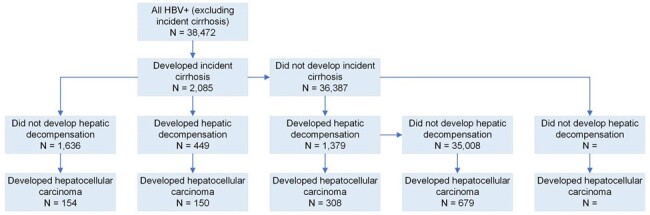

**Results:**

Of the 44,951 individuals with HBV, 62.9% were referred to gastroenterology, hepatology, or infectious disease specialist. Only 14.5% were initiated on an approved HBV treatment regimen. The mean time to treatment initiation was 34.1 months. Among 38,472 individuals without baseline cirrhosis, the incidence rates/100 person years (95% CI) for cirrhosis, HD, HCC, and death were 0.60 (0.58,0.63), 0.72 (0.69,0.75), 0.49 (0.47,0.52), and 4.29 (4.22,4.35) respectively.

**Conclusion:**

While the incidence rates for liver related complications and death are high among those with HBV infection, large gaps remain in linkage to care and treatment initiation. Terminal events, e.g. hepatic decompensation and HCC, can occur without antecedent cirrhosis, highlighting the need for early screening, diagnosis and treatment. Focused interventions are required to bridge these gaps in care.

**Disclosures:**

Adeel A. Butt, MD, MS, Gilead Sciences: Grant/Research Support

